# Correction: Expansion microscopy provides new insights into the cytoskeleton of malaria parasites including the conservation of a conoid

**DOI:** 10.1371/journal.pbio.3001401

**Published:** 2021-09-14

**Authors:** Eloïse Bertiaux, Aurélia C. Balestra, Lorène Bournonville, Vincent Louvel, Bohumil Maco, Dominique Soldati-Favre, Mathieu Brochet, Paul Guichard, Virginie Hamel

In the U-ExM resolves the hemispindles and reveals a distinct organisation of subpellicular microtubules in P. falciparum and P. berghei schizonts subsection of the Results, two references are omitted from the fifth sentence of the second paragraph.

The correct sentence is: To ascertain that the observed structures corresponded to subpellicular microtubules, we labelled proteins of expanded parasites in bulk with N-hydroxysuccinimide (NHS) ester-dye conjugates, which bind to amines as previously described (M’Saad et al, 2020 and Mao C et al, 2020) (S4 Fig).

The references are:

M’Saad O, Bewersdorf J. Light microscopy of proteins in their ultrastructural context. Nat Commun. 2020;11: 1–15. doi:10.1038/s41467-020-17523-8

Mao C, Lee MY, Jhan J-R, Halpern AR, Woodworth MA, Glaser AK, et al. Feature-rich covalent stains for super-resolution and cleared tissue fluorescence microscopy. Sci Adv. 2020;6: eaba4542. doi:10.1126/sciadv.aba4542
