# Supplementary figures and images for: Expansion microscopy provides new insights into the cytoskeleton of malaria parasites including the conservation of a conoid
Source: PLoS Biol. 2021 Mar 11;19(3):e3001020. doi: 10.1371/journal.pbio.3001020 (PMC7951857; doi:10.1371/journal.pbio.3001020)

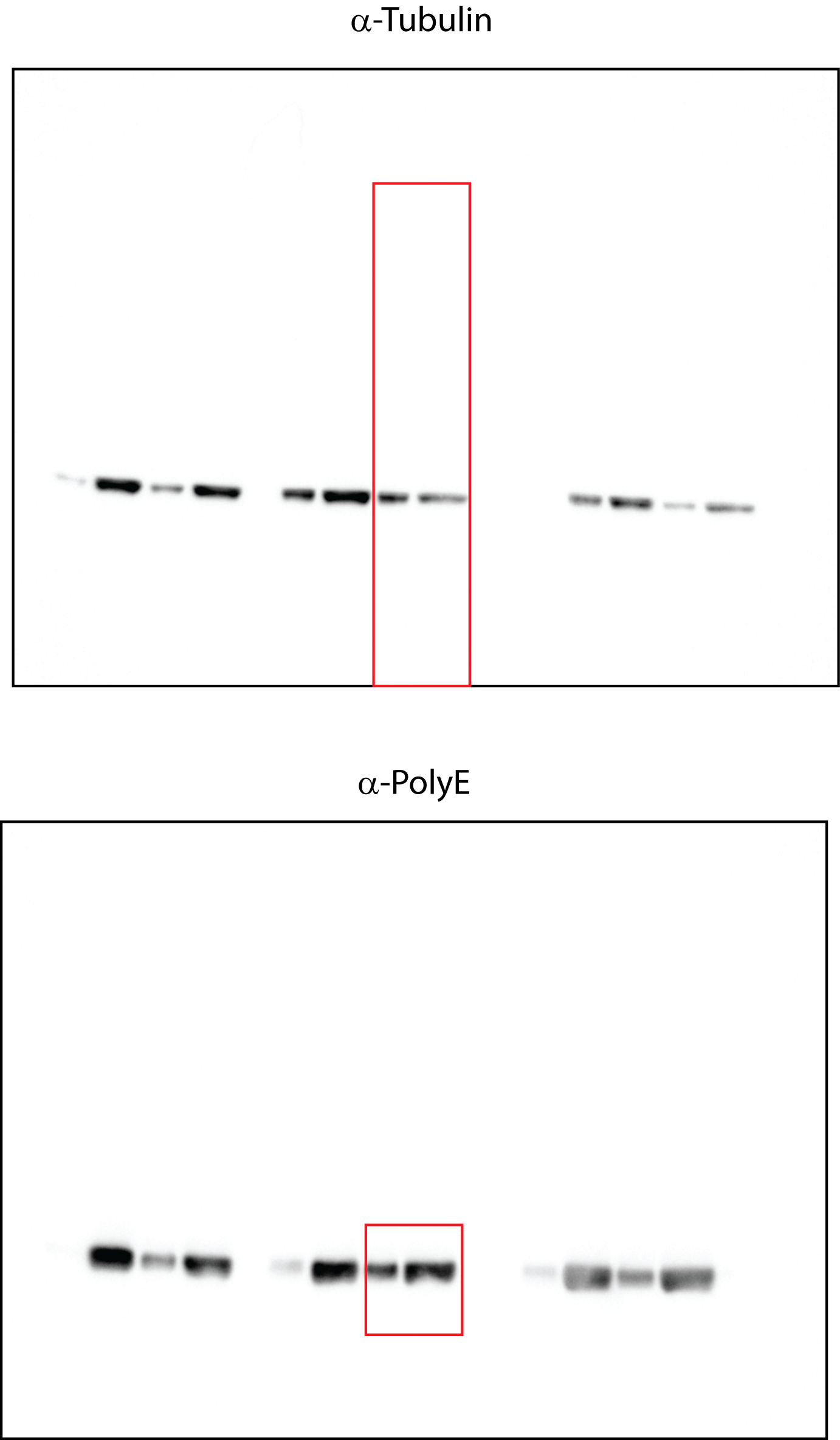

Supplement: S2 Data — (TIF) [file pbio.3001020.s002.tif]
