# Supplementary material for: Expansion microscopy provides new insights into the cytoskeleton of malaria parasites including the conservation of a conoid
Source: PLoS Biol. 2021 Mar 11;19(3):e3001020. doi: 10.1371/journal.pbio.3001020 (PMC7951857; doi:10.1371/journal.pbio.3001020)
Supplement: S1 Table — All cell lines as well as primary and secondary antibodies used in this study are listed, including the dilution used, the source or reference, and the catalogue number. (DOCX) [file pbio.3001020.s012.docx]

| **S1 table** | | | | |
| --- | --- | --- | --- | --- |
| **Reagent type or resource** | **Designation** | **Source or reference** | **Identifiers** | **Additional information** |
| Cell line  *P. berghei* | ANKA 2.34 | Billker et al, 2004 |  |  |
| Cell line  *P. berghei* | Kin8B-KO | Zeeshan et al, 2019 |  |  |
| Cell line  *P. berghei* | SAS6L-GFP | Wall et al, 2016 |  |  |
| Cell line  *P. berghei* | MyoB-GFP | Yusuf et al, 2015 |  |  |
| Cell line  *P. falciparum* | 3D7 | Walliker et al, 1987 |  |  |
| Antibody | Anti-GFP | Torrey Pines Biolabs | Cat#TP401 | U-ExM: 1:250 |
| Antibody | Anti-centrin from mouse (20H5) | Merck Millipore | Cat# 04-1624 | UExM: 1:300 |
| Antibody | α-tubulin  Mouse or Guinea pig | Unige antibody | AA345 | U-ExM: 1:250  IFA: 1:500 |
| Antibody | β-tubulin  Mouse or Guinea pig | Unige antibody | AA344 | U-ExM: 1:250  IFA: 1:500 |
| Antibody | PolyE | AdipoGen | AG-25B-0030 | U-ExM: 1:500  IFA: 1:1000 |
| Antibody | anti-mouse Alexa 488 | Eugene | A11008 | U-ExM: 1:400  IFA: 1:600 |
| Antibody | anti-mouse Alexa 568 | Eugene | A11004 | U-ExM: 1:400  IFA: 1:600 |
| Antibody | anti-rabbit Alexa 488 | Eugene |  | U-ExM: 1:400  IFA: 1:600 |
| Antibody | α-tubulin mouse | Sigma-Aldrich | T6199 | WB: 1:1000 |
| Antibody | anti-mouse HRP | Sigma-Aldrich | A5278 | WB: 1:3000 |
| Antibody | anti-guinea pig Alexa 568, | Eugene |  | U-ExM: 1:400 |
| Chemical | NHS Ester Dylight 488 | Thermofisher | 46402 | 10 μg/mL |
