# Supplementary material for: Expansion microscopy provides new insights into the cytoskeleton of malaria parasites including the conservation of a conoid
Source: PLoS Biol. 2021 Mar 11;19(3):e3001020. doi: 10.1371/journal.pbio.3001020 (PMC7951857; doi:10.1371/journal.pbio.3001020)
Supplement: Attachment — Submitted filename: 108700_1_rebuttal_1882459_qhjlvn.pdf [file pbio.3001020.s013.pdf]

Review Commons Referred Preprint RC-2020-00376

We would like to thank the editors and reviewers for their comments and suggestions to further improve the quality of our manuscript. In this letter, we provide answers and details addressing the issues raised by the reviewers. Please note that the modified text is highlighted in red to ease following-up the modifications within the revised manuscript.

Reviewer #2 (Evidence, reproducibility and clarity (Required)):

This paper describes the discovery of a new tubulin-based ring structure at the apical part of the highly polarized Plasmodium ookinete that has so far eluded scientists' scrutiny. The authors come to their conclusion by the use of expansion microscopy, which they apply for the first time (to my knowledge) to Plasmodium parasites. Their images are both stunning and beautiful and will be motivating many others to pursue expansion microscopy in these parasites. This paper hence presents both a technical advance as well as some interesting discovery of a new cytoskeletal structure in malaria parasites.

We thank the reviewer for their enthusiasm about our manuscript.

Major request:

Is it possible to distinguish between tubulin being a composite of the ring as a monomer or polymer? The authors could, for example, stain with SiR tubulin. Where is gamma-tubulin located?

We thank the reviewer for this interesting comment. However, to our knowledge, there is no way to discriminate monomeric tubulin versus polymerized tubulin at the ring. SiR-tubulin would have been the best option, however, it is not compatible with expansion microscopy as the sample is denatured at 95°C. Consistent with this, we could not observe any signal by staining expanded ookinetes with SiR-tubulin.

Instead, we performed immunofluorescence assays to distinguish between α and β tubulin and found that both are present at the ring, indicating that ATR is likely made of α-β heterodimers. This data now is included in the new Figure S6 of the revised manuscript.

We next stained ookinete for γ-tubulin using two different commercially available antibodies, respectively. However, no specific signal could be observed (see figure below). This was expected as in *Toxoplasma* tachyzoites, the apical polar ring does not contain γ-tubulin (PMID 25380753).

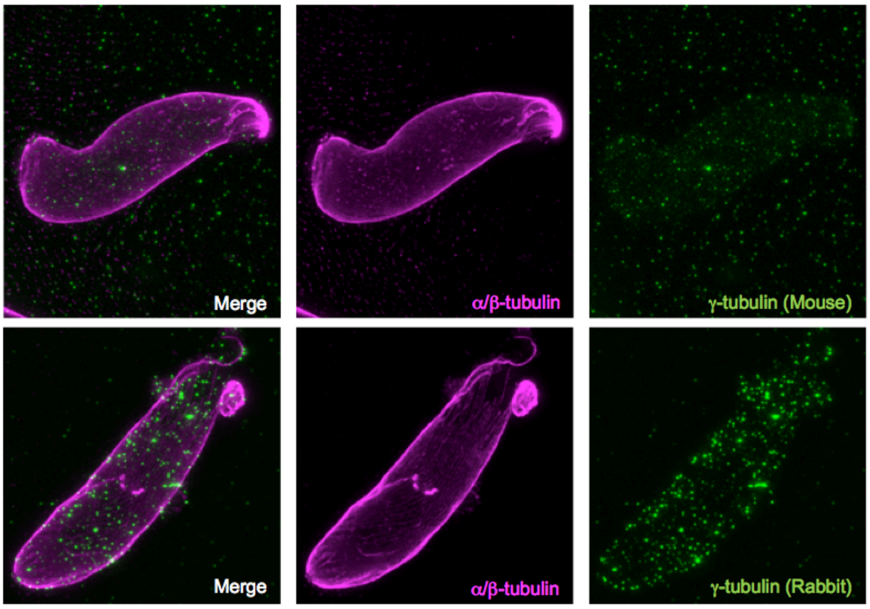

Minor points:

We thank the reviewer for pointing out these points that we corrected accordingly.

Abstract line 8: ookinetes

Corrected

Page 3: add references after sentences ending with invasion and cell; change 'if' to 'While' on last sentence

Added and changed

Page 4: add 'partly' after STED has; state somewhere that U-ExM has been performed already in T. gondii (two recent papers)

Done and citations added

Page 5: add 'length' in second last line

Added (page 6 of the revised manuscript)

Page 7: add reference at sentence ending with beating; change 'explosive' for 'rapid';

Added and changed

Page 7/8: can you distinguish singlet from doublets, e.g. by line plots?

We thank the reviewer for this question that is of great interest, however, we do not have the resolution to distinguish between singlets and doublets. Therefore, we

rephrased the sentence mentioning short microtubules instead of singlets or doublets (page 7 of the revised manuscript).

Page 8: please quantify 'seems more strongly'; 'We believe' - state on what basis you believe

We thank the reviewer for their comment. We have now clarified this point and show 3 examples of quantification of PolyE signal in axonemes and non-assembled axonemes (Figure S3). This result clearly established that fully assembled axonemes are more polyglutamylated than incomplete ones.

We have additionally performed a semi-quantitative analysis by western blot of tubulin and polyglutamylated tubulin in gametocytes 15 minutes post-activation. This revealed that both tubulin and polyglutamylated tubulin levels are similar between the wild type and the Kin8B-KO. This is now stated in page 8 of the revised manuscript: "Consistent with this, tubulin polyglutamylation was not affected in the mutant line (**Figure S3**)."

In addition, we rephrased the sentence starting by "we believe" by "Based on these results, we anticipate that U-ExM will reveal new aspects of *Plasmodium* gametogenesis that have been overlooked by conventional fluorescence microscopy" (page 9 of the revised manuscript).

Page 9: '2 to 3' should be 2 to 4 and is only true for merozoites. How many do ookinetes and sporozoites have?

We do observe two to three subpellicular microtubules in merozoites, a figure that is consistent with two previous references: 10.1111/cmi.12132 and 10.1016/j.molbiopara.2019.02.007

Ookinetes have around 60 subpellicular microtubules but this has not been exactly quantified (see below).

Sporozoites have 16 subpellicular microtubules, please see 15252/embj.2018100984, for example.

Page 10: over 40 microtubules: how does that compare to numbers determined by EM?; really no EM image out there in the old literature that describes a ring?

We are not aware of any rigorous quantification of the number of subpellicular microtubules in *Plasmodium* ookinetes. In reference 10.1128/MMBR.66.1.21-38.2002, the authors suggest that *Plasmodium* ookinetes have around 60 subpellicular microtubules, a number that is frequently cited in the literature. We looked for papers where transversal sections of ookinetes are shown and we counted 59 in 10.15252/embj.2019104168 (*P. berghei*), 47 in 10.4269/ajtmh.2010.10-0433 (*P. falciparum*), 59 in 10.1371/journal.pone.0201651 (*P. berghei*) and around 60 in 0.1111/cmi.12283 (*P. berghei*). We are now indicating this range in the result section (p.10 of the revised manuscript).

Looking back at previously published or unpublished EM images, it is possible to guess a tubular structure in line with the IMC this is however not obvious

as the structure is thinner and not as electron dense as the conoid in *Toxoplasma*. Deoxycholate extraction did also not reveal the ring, which was probably lost during the extraction. (10.15252/embj.2019104168).

Page 12: can you compare the curvature of the tubulin ring with that of other species and the conoid of *T. gondii*?

We are not able to visualize the fine structure of the tubulin ring but we now discuss this point in the discussion (p.13).

Page 13: Please discuss data from preprint by Waller and colleagues on localization in sporozoites; 'observation' -> 'observed'

We are now speculating in the discussion (p.14 of the revised manuscript), which of the proteins identified by Waller and colleagues may make the link between the APR and ATR. We are however not sure how to discuss the localization of these proteins in sporozoites as we have not imaged these proteins at this stage.

Changed

References 10 and 12 seem the same

We apologize for this mistake. The two references are now clearly distinct papers.

Figure 3 panel J: y-axis: length rather than size?

We thank you for noticing this mistake, we have corrected it accordingly.

Figure 4 legend: please use sensible digits e.g. 240+/-41 instead of '237.69' etc

We have simplified the values accordingly to the first digit, ie 237.69 becomes 238.

Figure 5: can you see the microtubule basket as described by Sinden and colleagues? Or are those the fan shaped microtubules?

We think that what we describe here correspond to the microtubule basket described by Sinden and colleagues. We are now using the two words to avoid any ambiguity.

Figure S2 legend: gametes

We have changed it accordingly.

Reviewer #2 (Significance (Required)):

The discovery of a novel cytoskeletal structure described in this paper should be of clear interest to all scientists studying molecular and cellular biology of malaria

parasites as well as other protozoologists and should be motivating for general cell biologists to study parasites. It is hard to compare this paper to others in the field. Some might be the localization and function of SAS6 in *Plasmodium* and *Toxoplasma* (deLeon Euk Cell 2012; doi: 10.1128/EC.00096-13; Marques et al. Cell Microbiol 2015; doi: 10.1111/cmi.12355 and Wall et al. Sci Rep 2016; doi: 10.1038/srep28604) although I find the current study more interesting due to the mix of new technology and new insights. Furthermore, the discovery of the *Toxoplasma* conoid as a queer microtubule-based structure (Hu et al., JCB 2002; doi: 10.1083/jcb.200112086) could be compared, although this might have appealed to a somewhat more general cell biology audience than the current paper due to the incredibly curious tubulin structure.

Again, we thank the reviewer for their supportive comments.

Reviewer #3 (Evidence, reproducibility and clarity (Required)):

In this work, Bertiaux et al. used expansion microscopy to investigate the tubulin-containing cytoskeletal structure of several different stages of Plasmodium parasite. The authors showed that expansion microscopy allows for visualizing fine features that are unresolvable with traditional light microscopy. Importantly, the structural details described here are consistent with what has been observed in the previous EM studies, which is an important validation of this approach. Overall the manuscript is well-written. The data are of high quality. There are only a few comments on the text that need to be addressed.

We thank the reviewer for their positive comments and we amended the manuscript according to the issues raised below.

1.Abstract- Page 2: related to the known function of the conoid, the authors stated that "This microtubule structure was presumed to be lost in *Plasmodium* despite its crucial role in both motility and invasion in most apicomplexans". This statement is not accurate. The function of the conoid has only been studied in *Toxoplasma gondii*, which is one, not "most" of the apicomplexans. Also in Toxoplasma, the conoid is formed of non-tubular tubulin polymers, not microtubules. It is more accurate to describe conoid as a tubulin-containing structure.

This is absolutely correct, we have changed the abstract accordingly

2.Page 8- "Polyglutamylation was observed on both assembling and full-length axonemes (Figure 2A-D), although we also observed that some microtubules that were not incorporated into the 9+2 pattern were lacking polyglutamylation" Expansion microcopy cannot resolve the 9+2 pattern of MTs in a flagellum. It is therefore more accurate to use "mature flagella" instead of "the 9+2 pattern" in this sentence.

We completely agree with the reviewer and corrected the sentence accordingly.

3.Page 8- typo "Altogether, U-ExM unveils details of axoneme formation in whole cells to levels that have only be accessible by EM." "have only be" should be written as "have only been"

We corrected the typo accordingly.

4.Figure 2F: I don't see how the location indicated by the open arrowhead could be a basal body position.

We understand the reviewer's comment. We inferred that was the position of a basal body owing to the PolyE signal that is always enriched at basal bodies. To make it clearer, we now position the open arrowheads in the PolyE channels as well.

5.Figure 3F legend: "(F) Histogram representing the number of hemispindles displaying 1, 2, 3, 4, 5, 6, 7, or 8 microtubules." What's the evidence that each linear structure is a single microtubule?

We agree with the reviewer that we cannot be absolutely sure that each linear structure is a single microtubule and not a bundle. We corrected the legend of Figure 3F with tubulin structures.

Reviewer #3 (Significance (Required)):

The implementation of expansion microscopy is significant for characterizing the cellular architecture of *Plasmodium*

Reviewer #4 (Evidence, reproducibility and clarity (Required)):

This manuscript describes the use of cutting-edge microscopy to not only show in great detail tubulin dynamics in multiple stages of *Plasmodium* parasites but also convincingly shows the possibility of *Plasmodium* parasites harbouring a conoid in at least the ookinete stage. It has until now largely been accepted that *Plasmodium* parasite had lost the conoid structure, and it is only through new exciting techniques such as expansion microscopy that allows for the identification of hard to find morphological details. This study is scientifically sound and the conclusions reached are well supported by the results presented.

We thank the reviewer for their enthusiasm about our manuscript and their positive comments.

Major Comments:

The authors focused mostly on *P. berghei* for the gametocyte and understandably so for ookinetes but there was no mention of *P. berghei* schizonts (or even free merozoites). While we understand the asexual stages were treated to focus on the sexual stages (as outlined in the methods), we feel the paper would be improved if at least this stage was included and if possible to also go further through the life cycle to include sporozoites (and as such all invasive forms where the conoid would be likely most important) as this is a clear benefit of using the *P. berghei* model as all stages

are relatively accessible. And while we think the addition of *P. falciparum* schizonts already included in the paper give vital insight, it would also be of interest to at least include *P. falciparum* gametocytes.

We understand the reviewer's point and have now included analysis of *P. berghei* schizonts and free merozoites in the manuscript (Figure 3 and Figure S4). This revealed a different number of subpellicular microtubules between *P. berghei* and *P. falciparum* merozoites further highlighting the diversity of the apical complex even within the same species. We however think that incorporating additional stages and species is not necessary to support the main claims of this manuscript.

Minor Comments:

1)at the bottom of page 3 it reads "If the structure of the apical complex is relatively well characterised..." - replace If with While.

We have corrected this sentence.

2)Figure 1: the description of ratio vs expansion factor is not in the figure legend, it appears in supplementary figure 1. This should be in the Figure 1 legend.

We apologize for this omission and have added it.

3)Page 7 the authors use to describe the proportion of cells either percent and ratio - 4% (1/25), just the ratio (20/25) or just the percent 44%. Please pick one way and use for all.

We apologize for this inconsistency and have chosen to indicate both information for all.

4)Considering MyoB is suggested to be expressed in schizonts it would also be of interest to look at this localisation with this line at schizonts stages (or in free merozoites)

Following the reviewer's suggestion, we have analyzed by expansion microscopy *P. berghei* schizonts expressing MyoB-GFP. However, the protein is not highly expressed at this stage and we have not been able to observe a MyoB-GFP signal following sample expansion.

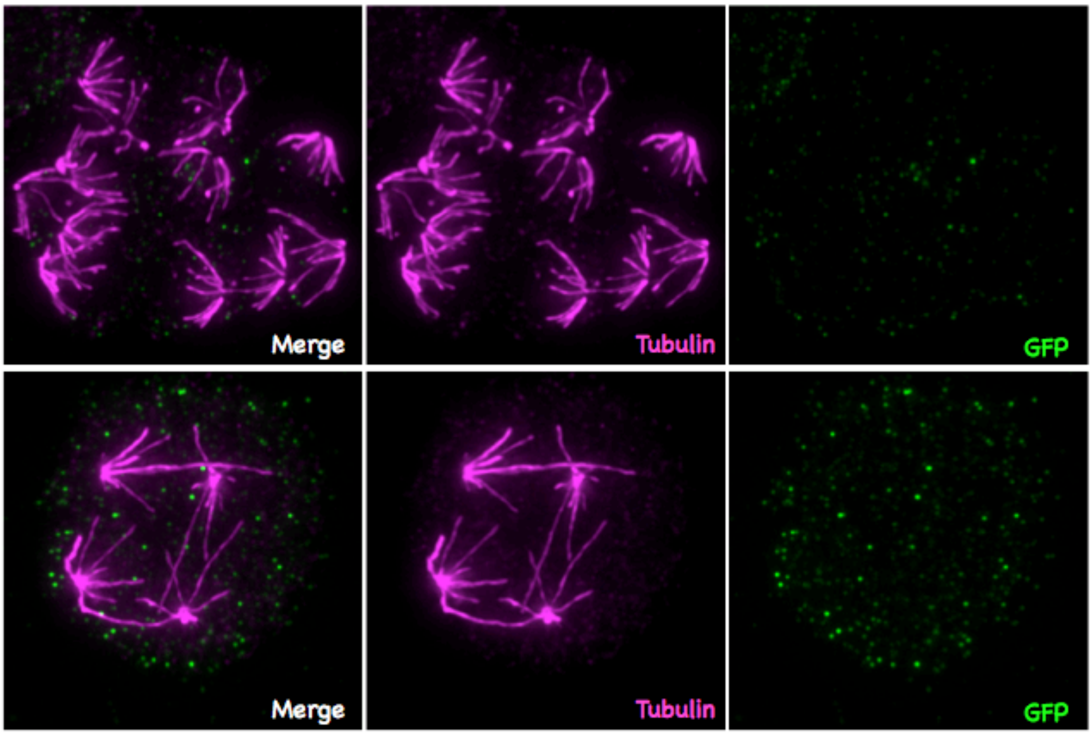

Reviewer #4 (Significance (Required)):

This paper not only clearly shows the benefits of the cutting-edge expansion microscopy technique it helps to change the current dogma on the presence of a conoid in *Plasmodium* parasites and as such is highly significant. This manuscript would have broad appeal not only to parasitologists but also to those with an interest in microscopy and cell biology.

Our expertise is largely in molecular parasitology and microscopy
